# Some Aspects of Intestinal Auto-Intoxication with Report of Two Cases Showing Unusual Skin Manifestations

**Published:** 1908-09

**Authors:** Lewis M. Gaines

**Affiliations:** Atlanta, Ga.


### Journal-Record of Medicine

Successor to Atlanta Medical and Surgical Journal, Established 1855, and Southern Medical Record, Established 1870.

**OWNED BY THE ATLANTA MEDICAL JOURNAL CO.**

**Published Monthly.**

*Official Organ Fulton County Medical Society, State Examining Board, Presbyterian Hospital, Etc.*

EDGAR G. BALLENGER, M. D., Editor.
BERNARD WOLFF, M. D., Supervising Editor.
A. W. STIRLING, M. D., C. M., D. P. H., J. S. HURT, B. Ph., M. D., Associate Editors
E. W. ALLEN, Business Manager.

COLLABORATORS

DR. W. F. WESTMORLAND, General Surgery.
F. W. McRAE, M. D., Abdominal Surgery.
H. F. HARRIS, M. D., Pathology and Bacteriology.
E. B. BLOCK, M. D., Diseases of the Nervous System.
MICHEL HOKE, M. D., Orthopedic Surgery.
CYRUS W. STRICKLER, M. D., Legal Medicine and Medical Legislation.
E. C. DAVIS, A. B., M. D., Obstetrics.
E. G. JONES, A. B., M. D., Gynecology.
R. T. DORSEY, Jr., B. S. M. D., Medicine.
L. M. GAINES, A. B., M. D., Internal Medicine.
J. N. LeCONTE, M. D., Diseases of the Stomach and Intestiness.
L. B. CLARKE, M. D., Pediatrics.
EDGAR PAULIN, M. D., Opsonic Medicine.
R. R. DALY, M. D., Medical Society.
A. W. STIRLING, M. D., Etc., Diseases of the Eye, Ear, Nose and Throat.
BERNARD WOLFF, M. D., Diseases of the Skin.
E. G. BALLENGER, M. D., Diseases of the Genito Urinary Organs.

VOL. X. SEPTEMBER 1908. NO. 6

### SOME ASPECTS OF INTESTINAL AUTO-INTOXICATION WITH REPORT OF TWO CASES SHOWING UNUSUAL SKIN MANIFESTATIONS.

BY LEWIS M. GAINES, A. B., M. D., ATLANTA, GA.

There is no longer any doubt that the existence of indican in the urine is a positive indication of the putrefaction of protein material in the intestine.[1] Furthermore, excepting gross pathological changes, such as obstruction, it is known that the greater amount of these putrefactive chances occur in the caecum, ascending colon and transverse colon. It is also established that indol is a product of the activity of B. Coli commune exerted upon either the products of proteolytic digestion, or upon substances formed by the action of facultative anaerobes present in the intestines.[2]

The indol thus formed may be to a greater or less extent absorbed, oxidized and conjugated with sulphuric acid finally to

(1) Ellinger—Zeit f. Physiol. Chemie, 1903, XXXIX, 44.
(2) Houghton—Am. Jour. Med. Sc., April, 1908.

be excreted in the urine as indoxyl sulphate (indican). Hence indican is the urinary form of indol, and indol is known to possess a degree of toxicity which varies according to the amount of its absorption, the ease of its oxidation to harmless indican, and probably according to individual idiocyncrasy, which may explain the diversity of clinical symptoms produced.

The appearance, then, of indican in the urine, we correctly assume, indicates intestinal putrefaction, and if we have associated with this certain clinical symptoms having no other positive basis, we may correctly diagnose intestinal auto-intoxication as the prominent, if not the sole cause, of the morbid condition. Chief among these clinical symptoms are nervous manifestations, such as headaches and certain forms of neurasthenia, cardio-vascular changes, muscular and joint symptoms,[3] eye symptoms,[4] skin lesions, and frequently the syndrome so often glibly explained by that fetish "uric acid diathesis."

The source and significance of indol being no longer a matter of dispute, there remains to investigate the conditions which promote its formation as well as those which influence its absorption, oxidation, and subsequent excretion.

These questions constitute the problems of metabolism which concern vitally our ability to relieve a large class of patients who in the past have been in great part treated symptomatically, and many times with indifferent success.

The formation of indol is controlled largely by two factors: first, the rapidity with which the products of proteolytic digestion are absorbed; second, bacterial activity. In regard to the first factor it has been shown that the potential indol content of a given protein may not be foretold except under given conditions affecting the rapidity of absorption of its split products, which determine whether or not the indol stage is reached. This fact bears the fruitful therepeutic lesson that we can regulate the diet so as to provide only those proteins most readily and most completely split by the proteolytic enzymes. Proteins slowly and incompletely split will naturally present in the intestine a residue consisting of a mixture of products in varying stages of hydrolytic cleavage, thus rendering absorption propostionately slow. Closely connected with this slow absorption is the matter of imperfect

(3) The work of Dr. Michael Hoke, of Atlanta, among others is convincing of the part played by auto-intoxication in joint pathology.

(4) G. E. DeSchweinitz—Jour. A. M. A., Vol. L, No. 25.

mastication of proteins, or the swallowing in bulk of milk. We may have a readily hydrolized protein but if the protein masses are large, the enzymes cannot reach their interior and a residue of great indol-producing capabilities result. Patients who complain that milk disagrees with them, frequently find they have no further trouble if they take it very slowly, as from a nipple, or by deliberately spending ten minutes in sipping one glassful.

The second factor in indol formation, bacterial activity, may to some extent be influenced by therapeutic measures. The B. Coli commune is a normal but not indispensable inhabitant of the colon, as was shown by Nuttall. Not only is this organism useless but it may easily become harmful whenever the protective mechanism of the body weakens. As shown above, one protective mechanism is rapid absorption of proteins, thus precluding an abundant protein pabulum for the micro-organisms. Another protective mechanism is the lactic acid formation which normally takes place in the small intestine in the digestion of carbohydrates. The acid reaction is inimical to the activity of all putrefactive organisms. This fact has, with profit, been taken advantage of in administering milk in which lactic acid fermentation has been brought about by introducing a pure culture of the lactic acid bacillus into the fresh milk, and incubating. A diet largely consisting of this sour milk has the double advantage of containing only easily absorbable protein, and of possessing this inhibiting power over protein putrefaction.

Although the diet is probably the principal desideratum, there are a group of drugs, which may also inhibit bacterial activity. These drugs do not kill the micro-organisms, but they do repress, to a certain extent, their activity. Of these drugs the most reliable are beta-naphthol, the salts of bismuth, and salol. I am confident I have gotten results from these drugs.

Concerning the factors which influence the absorption of indol we know but little. A large part of the indol formed in the intestine is excreted in the feces. It is furthermore a matter of uncertainty whether the normal mucosa can absorb an appreciable quantity of indol.[2] It is possible that the body not only takes precautions against its formation, but against its absorption as well. After absorption into the blood, oxidation of the indol takes place, followed by the conjugation with sulphuric acid thus changing the toxic substance into indican. This is a further protec-

(2) Houghton—Am. Jour. Med. Sc., April, 1908.

tive mechanism of the body. We know, however, that the oxidizing of the body varies. If low, the oxidation may be incomplete, the amount of indican in the urine slight and severe toxic symptoms manifested. Hence the absence of indicanuria is not a sign of the absence of putrefaction and of the activity of the toxic indol. On the other hand a marked indicanuria may mean excessive production of indol over and above the oxidizing power of the body, hence accompanied by toxic symptoms; or it may mean complete oxidization of the indol and hence no toxic symtoms.[1] Emerson[5] quotes a case with chronic constipation whose urine contained large quantities of indican for several years and yet who enjoyed the best of health. *A priori,* this is an example of complete oxidation. Should the oxidation fail, toxic symptoms would present themselves.

The practical conclusion to be drawn from these considerations is that the presence or absence of indican in the urine must be interpreted by the associated clinical symptoms and is of entirely different significance from the occurrence of other pathological constituents such as albumin. Jaffe[6] estimates the normal average amount of indican in the urine to be 6.6 mg. in 24 hours. Houghton,[2] however, maintains that this is incorrect and that there is normally no output as shown by the practical absence of indican in the urine of healthy children and of middle aged individuals under strict regimen. Emerson[5] states that 5.25 mg. is normally present in 24 hours. It has been rare in my experience to find an absence of indican, but whether or not the presence of even a small amount is normal or not, is open to question. As shown above, the absence of symptoms in cases of indicanuria may be explained by the high oxidizing power, or as Houghton further suggests because indol absorption need not extend over a long enough period for the appearance of toxic symptoms.

A not infrequent manifestation of intestinal intoxication is the appearance of various skin lesions. The following are two illustrative cases which have recently come under my obervation:

*Case* 1.—Mr. H., age 21, good family history, rheumatic past history. For six or seven years with the recurring advent of hot weather the backs of both hands became livid in appearance and there was a certain amount of itching. He had had verious diagnoise made upon him from eczema to Raynaud's Disease. At the

(5) Emerson—Clinical diagnosis, 1st Ed. p. 139.
(6) Jaffe —Pfluger's Archiv., 1870 iii, 448.
(2) Houghton—Am. Jour. Med. Sc., April, 1908.

time I saw him the last named diagnosis was in vogue and he had been taking arsenic for a number of months. The discoloration was accompanied by slight swelling and was more pronounced the hotter the weather. For the past six or seven years it had persisted all during the summer months entirely unaffected by local or general treatment.

*Examination.*—Well developed and nourished young man, all of whose organs appeared sound on physical examination. The only point of departure from normal was the local condition. Over the entire dorsum of both hands there was a purple discoloration which faded away toward the margins to a reddish-purple, and finally to a normal flesh tint. There was no eruption of the skin, but apparently a marked venous stasis in the capillaries, for on pressure the purple color disappeared and was succeeded by a blanched area which resumed the former purple tint when pressure was removed, thus excluding purpura. There was no pain on manipulation.

The urine of the patient showed nothing abnormal except a most intense indican reaction. This gave evidence of intestinal putrefaction and so treatment was directed accordingly. The alimentary canal was cleansed and then kept clean, and intestinal antiseptics administered and a diet containing sour milk advised. The change was remarkable. In one week nearly all trace of the trouble had disappeared and in ten days the patient was perfectly well.

*Case 2.*—Mr. W., age 20, came to me complaining of a painful area over the buttocks. His family history and past history were negative except for a tendency to constipation. Physical examination revealed nothing amiss except the local condition over the right buttock. Here an area as large as a man's hand was swollen uniformly, the skin being hot and red and the surface elevated above that of the surrounding skin by at least 1-8 inch. The borders of this inflamed area were remarkably clearly defined. The appearance suggested erysipelas, but there were no general symptoms, pulse and temperature being normal and the patient feeling perfectly well. The local condition, however, caused intense burning and itching so that he was in misery constantly and unable to sit. Eamination of the urine revealed an intense grade of indicanuria. The same general treatment was pursued as in Case 1, the bowels being thoroughly cleansed and intestinal antiseptics administered. In 24 hours there was great relief, and by the third day the buttock had resumed its normal condition, except for a slight discoloration of the skin.

*Conclusions.*—The general conclusion from the above discussion and illustrative cases may be very briefly drawn. In the present light of our knowledge the types of clinical symptoms above designated accompanied by indicanuria indicate the necessity of investigating the protein metabolism of the individual. If the careful supervision of the protein ingestion and the cleansing and rendering antiseptic the alimentary tract is followed by an ameliorization or disappearance of the symptoms, we can then justly conclude we had to do with intestinal auto-intoxication and that once again rational therapeutics is firmly intrenched.

822 Candler Building.

